# A DVLA Notification Audit in Forensic Supported Accommodation

**DOI:** 10.1192/bjo.2022.493

**Published:** 2022-06-20

**Authors:** Chung Mun Alice Lin, Nicole Lim, Neeti Sud

**Affiliations:** 1 National Institute of Health Research Newcastle Biomedical Research Centre and the Translational and Clinical Research Institute, Newcastle University, Newcastle upon Tyne, United Kingdom.; 2 Newcastle University, Newcastle upon Tyne, United Kingdom; 3 Cumbria, Northumberland, Tyne and Wear NHS Foundation Trust, Newcastle upon Tyne, United Kingdom

## Abstract

**Aims:**

The Driver and Vehicle Licensing Agency (DVLA) in England, Scotland and Wales are legally responsible for deciding if a person is medically unfit to drive. This means they need to know if a person holding a driving licence has a condition or is undergoing treatment that may now, or in the future, affect their safety as a driver. The driver is legally responsible for telling the DVLA about any such condition or treatment. Doctors should therefore alert patients to conditions and treatments that might affect their ability to drive and remind them of their duty to tell the appropriate agency. Patients with acute schizophrenia or an acute psychotic disorder must not drive and must notify the DVLA. In alliance with the above, the GMC advises that clinicians have a responsibility to explain the above information to the patient and inform them that they have a legal duty to inform the DVLA. Doctors should also inform patients that relevant medical information may need disclosing about them to the DVLA if they continue to drive against advice, and any advice given should be documented. The main objective of this audit is to identify if notification of DVLA for forensic patients living in supported accommodation, is in accordance with the DVLA guidelines.

**Methods:**

A total of 12 residents living in community forensic supported accommodation who have a notifiable diagnosis were included. Data collection took place in September 2021, looking through all previous records relating to the search words “DVLA”, “drive”, “driving” and “license”. Data audited were from the trust's electronic patient records.

**Results:**

Diagnoses included paranoid schizophrenia, delusional disorder and personality disorder. Antipsychotic medications included Olanzapine (oral and IM), Clozapine and Zuclopenthixol +/- antidepressants. Legal status included community treatment orders (civil section), voluntary community patients and those on a conditionally discharged restriction under secretary of State supervision. Two patients held full driving licences and a further two held provisional licences, with DVLA documented discussions and notification compliance at 100%. The remaining eight patients had no documentation regarding driving nor DVLA discussions or notification.

**Conclusion:**

This audit found that DVLA discussions are not currently well documented, with only four patient records that have this recorded. Although it is the clinical team's responsibility to advise the patient to notify the DVLA, it is ultimately the patient's responsibility to notify the DVLA themselves. DVLA discussions need to be had regardless of driving status and documentation should reflect this.

